# Influence of CYP2C8*3 and *ABCG2* C421A genetic polymorphisms on trough concentration and molecular response of imatinib in Egyptian patients with chronic myeloid leukemia

**DOI:** 10.1007/s00280-024-04723-y

**Published:** 2024-12-23

**Authors:** Safwat A. Mangoura, Mahmoud H. Abdel-Raheem, Hanan A. Eltyb, Mohammed S. Molla, Abeer M. R. Hussein

**Affiliations:** 1 Department of Pharmacology and Toxicology, Faculty of Pharmacy, Badr University in Cairo (BUC), Badr, Cairo, Egypt; 2 Department of Medical Pharmacology, Faculty of Medicine, Assiut University, Assiut, 71515 Egypt; 3 Department of Medical Oncology and Malignant Hematology, South Egypt Cancer Institute (SECI), Assiut University, Assiut, Egypt

**Keywords:** CML, Imatinib, CYP2C8*3, *ABCG2*

## Abstract

**Purpose:**

The treatment landscape for chronic myeloid leukemia (CML) has been revolutionized by the introduction of imatinib, a tyrosine kinase inhibitor, which has transformed the disease from a fatal condition into a manageable chronic illness for a substantial number of patients. Despite this, some individuals do not respond adequately to the treatment, and others may experience disease progression even with continued therapy. This study examined how CYP2C8*3 (G416A; rs11572080) and *ABCG2* C421A (rs2231142) single nucleotide polymorphisms (SNPs) affect the plasma trough concentration and therapeutic response of imatinib in Egyptian CML patients.

**Methods:**

The study included fifty patients with chronic-phase CML, who were categorized into two groups: responders (*n* = 26) and non-responders (*n* = 24), according to their *BCR-ABL1* transcription levels after 12 months of imatinib treatment. Genotyping of the CYP2C8*3 and *ABCG2* C421A polymorphisms was performed using polymerase chain reaction-restriction fragment length polymorphism (PCR-RFLP), while plasma trough concentrations were determined through high-performance liquid chromatography with ultraviolet-diode array detection (HPLC-UV/DAD).

**Results:**

Patients with the CA genotype of *ABCG2* C421A showed significantly higher mean plasma trough concentrations of imatinib (CA: 1731 ± 424.7 ng/mL; CC: 1294 ± 381.3 ng/mL; *p* = 0.0132) and demonstrated a better molecular response compared to those with the CC genotype (*p* = 0.0395).

**Conclusion:**

The *ABCG2* C421A polymorphism significantly influenced imatinib plasma trough concentrations and molecular responses in Egyptian chronic-phase CML patients. Genotyping of this polymorphism in these patients could assist in optimizing imatinib therapy, predicting more favorable treatment outcomes, and enabling the development of more personalized treatment plans.

**Supplementary Information:**

The online version contains supplementary material available at 10.1007/s00280-024-04723-y.

## Introduction

Chronic myeloid leukemia (CML) is a malignant tumor that results from the abnormal proliferation of hematopoietic stem cells within the bone marrow. It constitutes approximately 15% of leukemia cases in adults [1].

A hallmark of this disease is the “Philadelphia (Ph) chromosome”, which is produced by a balanced reciprocal translocation between the long arms of chromosomes 9 and 22 [t (9; 22) (q34; q11)] [2].

This genetic translocation transposes the *Abelson murine leukemia 1 (ABL1)* gene from chromosome 9 to the *breakpoint cluster region (BCR)* gene on chromosome 22, resulting in the generation of the *BCR-ABL1* fusion oncogene that encodes the BCR-ABL1 oncoprotein [3].

The treatment of CML was revolutionized in 2001 with the introduction of imatinib, a tyrosine kinase inhibitor (TKI). This advancement transformed CML from a life-threatening illness into a manageable condition for most patients [4, 5].

Despite this significant improvement in CML outcomes achieved with imatinib, some patients either experience disease progression during therapy or fail to respond adequately to the treatment [6].

Treatment failure with imatinib can be attributed to several mechanisms, including the upregulation of *BCR-ABL1*, the appearance of additional cytogenetic abnormalities, and mutations within the kinase domain of *BCR-ABL1* [7]. Furthermore, variability in imatinib pharmacokinetics could influence its efficacy, as a correlation between imatinib exposure and clinical outcomes has been demonstrated [8–10].

Imatinib exhibits significant inter-patient variability in trough concentrations [11]. This variability may be attributed to differences in drug-metabolizing enzyme activity, the function of influx/efflux transporters [12], interaction with other drugs [13], and patient non-adherence [14].

It is predominantly metabolized into N-desmethyl imatinib (NDMI), by cytochrome P450 (CYP) 3A4 isozyme [15]. Furthermore, CYP2C8 is integral to imatinib metabolism, especially when CYP3A4 experiences auto-inhibition at imatinib’s steady state [16, 17].

Other isozymes, including CYP1A2, CYP2D6, CYP2C9 and CYP2C19, also contribute to imatinib metabolism, but their roles are relatively minor [18].

Active efflux of imatinib from cells is carried out by adenosine triphosphate-binding cassette (ABC) transporters, notably ABCB1 and ABCG2. Conversely, the uptake of imatinib into cells is facilitated by the human organic cation transporter-1 (OCT1), also known as solute carrier family 22 member 1 (SLC22A1) [19].

Thus, genetic polymorphisms in the *CYP2C8* and *ABCG2* genes are likely to affect intracellular or systemic concentrations of imatinib, potentially altering its therapeutic efficacy.

A notable gap in research existed regarding these polymorphisms in Egyptian CML patients. Therefore, this study aims to evaluate the influence of CYP2C8*3 (G416A; rs11572080) and *ABCG2* C421A (rs2231142) single nucleotide polymorphisms (SNPs) on plasma trough concentration and therapeutic response to imatinib in Egyptian patients with CML.

## Materials and methods

### Study design

This observational, cross-sectional study was conducted at the Medical Oncology Department of the South Egypt Cancer Institute (SECI), Assiut, Egypt.

The study protocol was reviewed and approved by the Ethics Committee of the Faculty of Medicine at Assuit University, Egypt (Institutional Review Board number: 17200117) and was registered in the ClinicalTrials.gov database (Identification Number: NCT03262974).

### Chemicals and kits

GeneJet Whole Blood Genomic DNA Purification Mini Kit (Thermo Scientific, Lithuania), COSMO PCR Master Mix (Willowfort, England), Forward and reverse primers (Macrogen, South Korea), Restriction enzymes: *Bse*RI and *Hpy*CH4III (New England Biolabs, USA), Gel loading dye purple (6X) (New England Biolabs, USA), DNA ladder (GeneDireX, Taiwan) were purchased. Agarose powder (Bioline, USA), Tris base, Boric acid, ethylenediaminetetraacetic acid (EDTA) and ethidium bromide (Sigma-Aldrich, USA).

Imatinib mesylate (CAS No. 220127-57-1) and propranolol hydrochloride (CAS No. 318-98-9, the internal standard) were purchased from AK Scientific, Inc., USA. Acetonitrile and methanol (Sigma-Aldrich, Germany) were of HPLC grade. Water was purified by a Milli-Q Gradient A10 water purification system (Merck Millipore, USA).

### Patients

Patients were enrolled from the outpatient clinic of the Medical Oncology Department at the SECI, Egypt. The included patients had a diagnosis of Ph chromosome-positive CML, were aged over 18 years, and had been treated with imatinib for at least 12 months with good compliance to treatment. Exclusion criteria encompassed patients in accelerated or blastic phases, those taking medications that induce or inhibit liver microsomal enzymes (e.g., Ketoconazole, Phenytoin, and Valproic acid), individuals with poor compliance, and pregnant women. All patients provided informed consent before participation in the study.

Patient data, including age, sex, age at CML diagnosis, and *BCR-ABL1* transcript levels at 12 months post-start of imatinib treatment, were collected during their regular follow-up visits.

The molecular response to imatinib was assessed using the International Scale, which quantifies the ratio of *BCR-ABL1* transcripts to *ABL1* transcripts, expressed as a percentage of *BCR-ABL1* on a logarithmic scale. This scale corresponds to reductions of 2, 3, 4, 4.5, and 5 (equating to 1%, 0.1%, 0.01%, 0.0032%, and 0.001%, respectively) relative to the standardized baseline established in the International Randomized Study of Interferon versus STI571 (IRIS) [20].

In accordance with the European LeukemiaNet recommendations for CML treatment, treatment failure is defined as *BCR-ABL1* levels exceeding 1% after 12 months of imatinib therapy [21].

Accordingly, patients in this study were classified into two groups based on their *BCR-ABL1* transcript levels 12 months after initiating imatinib treatment: responders, with transcript levels of 1% or below, who continued imatinib therapy, and non-responders, with transcript levels above 1%, who were transitioned to second-generation TKIs such as nilotinib or dasatinib.

### Blood sampling

Peripheral blood samples were collected from patients into EDTA tubes during their routine follow-up visits at the outpatient clinic, concurrent with their regular laboratory tests.

Two milliliters were collected from all patients for DNA extraction and subsequent genotyping. An additional two milliliters were collected from patients receiving imatinib for at least 12 months, approximately 24 ± 3 h after their last dose but before the next dose (trough sample), following 28 consecutive days of administration [9].

Samples were then centrifuged at 2500 RPM (1000 ×*g*) for 15 min [22] using a benchtop centrifuge (Rotofix 32 A, Hettich, Germany). Plasma samples obtained were stored at -80 °C until subsequent measurement of trough concentrations.

All blood samples were collected within the same timeframe; however, there was a lapse between the molecular response evaluation and the blood sample collection for each patient, which depended on the duration of their imatinib treatment.

### DNA extraction and genotyping

Whole blood DNA was extracted using a commercial extraction kit following the manufacturer’s instructions. The concentration and purity of the extracted DNA were assessed using a microplate spectrophotometer (Epoch, BioTek Instruments Inc., USA). Ratios of A_260_/A_280_ ranging from 1.7 to 1.9 indicate pure template DNA, optimal for PCR [23]. Extracted DNA samples were stored at -80 °C until genotyping analysis.

The CYP2C8*3 (G416A; rs11572080) [24] and *ABCG2* C421A (rs2231142) [25] polymorphisms were genotyped using the polymerase chain reaction-restriction fragment length polymorphism (PCR-RFLP) method, as previously described. Detailed primer sequences, PCR thermal conditions, and restriction enzymes used are provided in the Online Resource 1.

The PCR was performed using a thermal cycler (Veriti 96 Well Thermal Cycler, Applied Biosystems, USA). The resulting PCR products were then digested with appropriate restriction enzymes according to the manufacturer’s protocol.

The digested products were separated by electrophoresis on 3% agarose gel using a horizontal electrophoresis unit (Biometra Compact M, Analytik Jena, Germany) and visualized under ultraviolet (UV) light at 312 nm wavelength using a UV transilluminator (UVstar, Analytik Jena, Germany). Gel images were captured and documented using a gel documentation system (BioDocAnalyze, Analytik Jena, Germany). DNA extraction and PCR-RFLP analyses were carried out at the Medical Research Center, Assiut University, Assiut, Egypt.

### Measurement of plasma trough concentration of imatinib

Plasma trough concentrations were measured using the high-performance liquid chromatography with ultraviolet-diode array detection (HPLC-UV/DAD) method described previously [26]. The analysis was performed using a Waters 2695 Alliance Separations Module equipped with a 996 PDA detector (Waters Corporation, USA), with the detector wavelength set at 265 nm. Chromatographic separation was achieved on a C18 reversed-phase analytical column (Inertsil ODS 4 analytical column, 250 mm × 4.6 mm internal diameter, five µm particle size; GL Sciences, Japan) using isocratic elution. Chromatogram processing, data generation, and concentration calculations were conducted using Empower 3 chromatography data software (Waters Corporation, USA). These measurements were carried out at Nawah Scientific Inc., Cairo, Egypt.

### Statistical analysis

Possible deviation from Hardy–Weinberg equilibrium (HWE) of various genotypes was assessed using Chi-square test.

The Shapiro–Wilk test was employed to evaluate the normality of variable distributions. Continuous variables that followed a normal distribution were reported as mean ± standard deviation (SD), whereas those deviating from normality were presented as median and range. Categorical variables were reported in terms of frequency and percentage.

Fisher’s exact test was utilized to compare the frequencies of various genotypes between imatinib responders and non-responders. Additionally, the unpaired Student’s *t*-test was applied to assess differences in plasma trough concentrations of imatinib across patients with different genotypes.

GraphPad Prism software version 9.5.1 (GraphPad Software Inc., USA) was used to perform statistical analyses and create graphical representations. Statistically significant differences were indicated by a *p*-value of less than 0.05.

## Results

### Patients’ characteristics

Table 1 presents the demographic and clinical characteristics of the patients. A total of 50 Egyptian patients with Ph chromosome-positive CML in the chronic phase were included in the study. Of whom, 30 (60%) were females. The mean age of the patients at enrollment was 43.84 ± 13.30 years. At the time of CML diagnosis, the mean age was 38.48 ± 13.25 years. Twenty-six patients (52%) were responders to imatinib, with a mean treatment duration of 5.58 ± 2.16 years. The remaining 24 patients (48%) were non-responders.

**Table 1:** Demographic and clinical characteristics of Egyptian patients with CML (*n* = 50)

**Sex:**	
- Male, *n* (%) - Female, *n* (%)	20 (40%) 30 (60%)
**Age** (years), mean ± SD	43.84 ± 13.30
**Age at diagnosis** (years), mean ± SD	38.48 ± 13.25
**Molecular response to imatinib at 12 months**:	
- Responders (*BCR-ABL1* transcript level ≤ 1%), *n* (%) - Non-responders (*BCR-ABL1* transcript level > 1%), *n* (%)	26 (52%) 24 (48%)
**Duration of treatment with imatinib** (years), mean ± SD	5.58 ± 2.16
***BCR-ABL1*transcript level at 12 month** (%):	
- Responders, median (range) - Non-responders, median (range)	0.2 (0-0.9) 30 (2-100)

### Genotype and allele frequency

Table 2 presents the frequency of different genotypes and alleles of CYP2C8*3 G416A and *ABCG2* C421A polymorphisms in our patients. For the CYP2C8*3 G416A polymorphism, the frequencies of the GG (CYP2C8*1/*1, homozygous wild type) and GA (CYP2C8*1/*3, heterozygous type) genotypes were 76% and 24%, respectively. The frequency of the variant allele (A allele) was 12%. For the *ABCG2* C421A polymorphism, the frequencies of the CC (homozygous wild) and CA (heterozygous) genotypes was 78% and 22%, respectively. The frequency of the variant allele (A allele) was 11%. In the present study, we did not detect the AA (homozygous variant) genotype for either CYP2C8*3 G416A or *ABCG2* C421A polymorphisms. All reported frequencies did not significantly deviate from the HWE (*p >* 0.05).

**Table 2:** Genotype and allele frequencies of CYP2C8*3 and *ABCG2* C421A polymorphisms among Egyptian patients with CML

SNP	Genotype	Frequency (%)	Allele	Frequency (%)	HWE *p*-value
CYP2C8*3 (G416A; rs11572080)	GG	76	G	88	0.3349
	GA	24	A	12	
*ABCG2* C421A (rs2231142)	CC	78	C	89	0.3821
	CA	22	A	11	

### Influence of different genotypes on imatinib trough concentrations

**Fig. 1:**
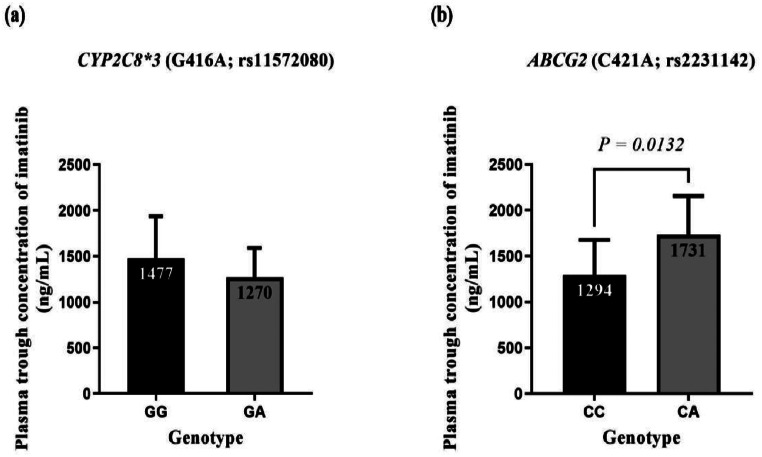
Plasma trough concentrations of imatinib in different genotypes of CYP2C8*3 (**a**) and *ABCG2* C421A (**b**) polymorphisms in CML patients

It was observed that there was no statistically significant difference in the mean plasma trough concentration of imatinib in patients carrying different genotypes of the CYP2C8*3 polymorphism (GG: 1477 ± 459.8 vs. GA: 1270 ± 321.5; *p* = 0.3995) (Fig. 1a).

On the other hand, patients carrying the CA genotype of the *ABCG2* C421A polymorphism had a statistically significant higher mean plasma trough concentration of imatinib compared to those carrying the CC genotype (CA: 1731 ± 424.7 ng/mL vs. CC: 1294 ± 381.3 ng/mL; *p* = 0.0132) (Fig. 1b).

Data are presented as mean ± SD. No statistically significant difference was found in the mean plasma trough concentration of imatinib between the different genotypes of CYP2C8*3 polymorphism (*p* = 0.3995). A statistically significant difference was observed in the mean plasma trough concentration of imatinib between the CC and CA genotypes of *ABCG2* C421A polymorphism (*p* = 0.0132). Results were compared using the unpaired Student’s *t*-test.

### Influence of different genotypes on molecular response of imatinib

**Fig. 2:**
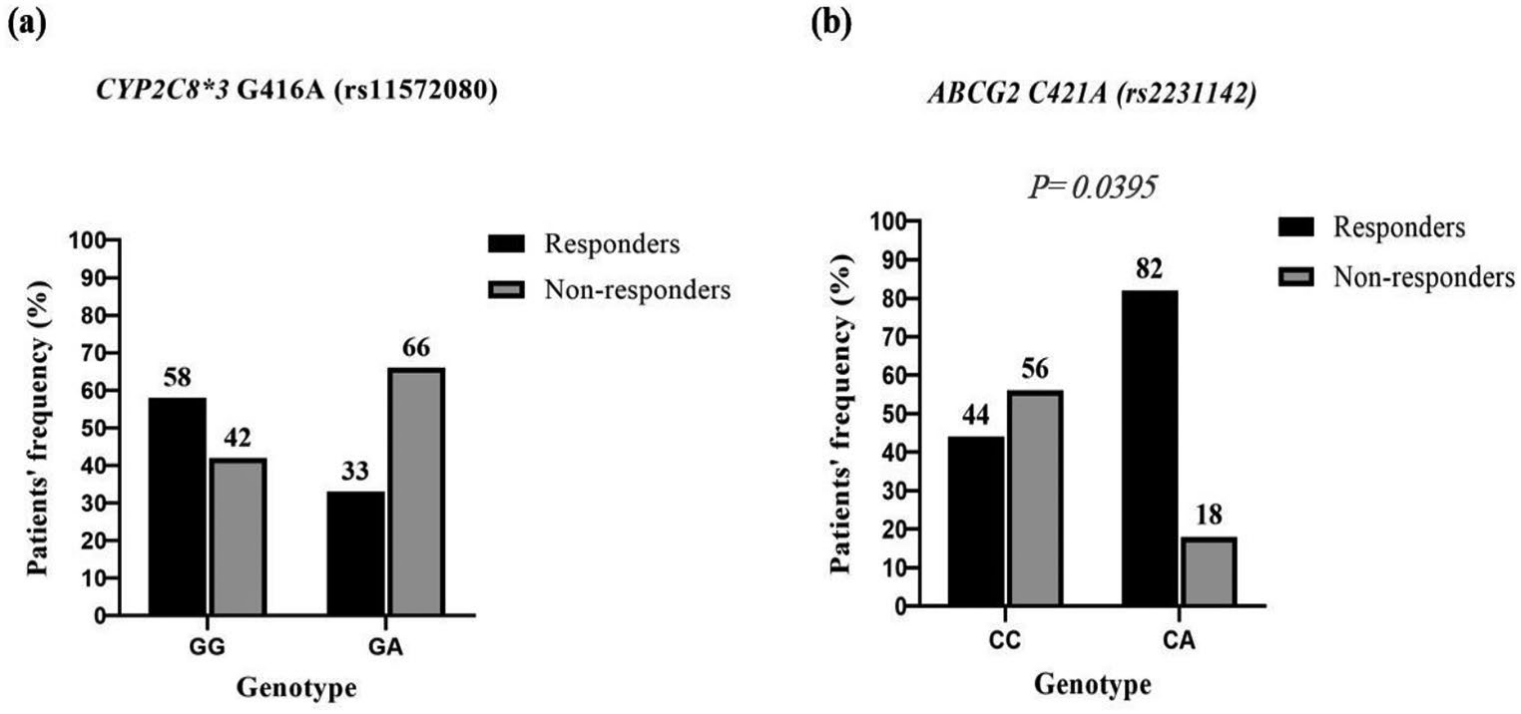
Frequency of different genotypes of CYP2C8*3 (**a**) and *ABCG2* C421A (**b**) polymorphisms in imatinib responders and non-responders

Regarding the genotypes of the CYP2C8*3 polymorphism, no statistically significant difference was observed in their distribution between responder and non-responder patients (*p* = 0.1902) (Fig. 2a).

However, a statistically significant difference was noted between the two patient groups regarding the distribution of different genotypes of the *ABCG2* C421A polymorphism with predominance of the CA genotype in responder patients (*p* = 0.0395) (Fig. 2b).

Assessment of molecular response to imatinib based on the *BCR-ABL1* transcript level at 12 months. Responders (*n* = 26); Non-responders (*n* = 24). No statistically significant difference was found between the frequency of GG and GA genotypes of CYP2C8*3 in the two groups (*p* = 0.1902). However, There was a statistically significant difference between the frequency of CC and CA genotypes of *ABCG2* C421A in the two groups (*p* = 0.0395). Results were compared using Fisher’s exact test.

## **Discussion**

Despite the transformative impact of imatinib on the treatment of CML, a substantial proportion of CML patients (30–35%) exhibit inadequate response, either as suboptimal responders or as resistant to the treatment [27]. Various factors contribute to these outcomes [28], including genetic polymorphisms that impact drug metabolism and transport [29].

In this study, we focused on two key SNPs, CYP2C8*3 (G416A; rs11572080) and *ABCG2* C421A (rs2231142), which may influence imatinib pharmacokinetics. These genetic variations can alter plasma trough concentrations of imatinib and impact treatment outcomes in Egyptian patients with chronic-phase CML.

Over the past three decades, many methods have been developed for SNP genotyping, with PCR-RFLP being a widely used and cost-effective option [30]. In this study, we utilized PCR-RFLP to genotype target SNPs in all 50 patients. However, this technique has limitations, including its dependence on specific restriction enzymes to distinguish alleles, which can restrict its effectiveness when suitable enzymes are unavailable or show non-specific binding. Additionally, the multi-step process is time-intensive, increasing the risk of contamination and error, and is less efficient than high-throughput methods like real-time PCR and next-generation sequencing [31].

Many authors recommend HPLC with UV detection for measuring imatinib plasma concentrations, as it is cost-effective, straightforward, and suitable for routine laboratory use. Studies have also shown that HPLC-UV provides results comparable to those obtained with LC-MS/MS [32, 33]. Additionally, incorporating a diode-array detector (DAD) into HPLC-UV systems improves specificity by allowing spectral comparisons and peak purity verification, making HPLC-UV/DAD sufficiently sensitive and specific for imatinib analysis [34].

The CYP2C8 enzyme, comprising 7% of the liver’s CYP content, is crucial for metabolizing about 20% of commonly prescribed medications. It is encoded by the *CYP2C8* gene, situated on chromosome 10q24 [35].

This isozyme shows significant genetic diversity, with various SNPs identified, including CYP2C8*2, *3, and *4, which account for most of the non-synonymous variability of *CYP2C8* in humans [36, 37].

Among these SNPs, CYP2C8*3 stands out as the most extensively studied functional polymorphism. It encompasses two non-synonymous variants, G416A (Arg139Lys; rs11572080) and A1196G (Lys399Arg; rs10509681), often found in complete or near-complete linkage disequilibrium [38].

In vitro studies suggest that CYP2C8*3 is linked to enhanced enzyme activity, which may expedite the conversion of imatinib to NDMI [11, 39].

However, in vivo studies indicate substrate-specific effects, including enhanced metabolism of drugs like pioglitazone and repaglinide, and reduced metabolism of ibuprofen [40].

In the context of our study on Egyptian CML patients, we did not observe a significant influence of the CYP2C8*3 polymorphism on imatinib’s plasma trough concentration or molecular response.

Nonetheless, we noted a trend towards lower imatinib concentrations in patients carrying the GA (CYP2C*1/*3) genotype, particularly prevalent among non-responders. These findings suggest a potential detrimental impact of this genotype on imatinib pharmacokinetics and clinical efficacy.

To our knowledge, this study is the first to investigate the impact of CYP2C8*3 (G416A; rs11572080) on imatinib therapy outcomes in Egyptian CML patients, highlighting the need for further validation through larger studies.

Results of the present study align with those of Verboom et al. [41], who similarly found no significant effect of the CYP2C8*3 on imatinib pharmacokinetics in Dutch patients with GIST or CML.

Conversely, studies by Barratt et al. [42] and Dalle Fratte et al. [43] reported increased metabolic ratios of NDMI/imatinib in CML and GIST patients carrying the CYP2C8*3 allele, contrasting with our findings. Further research in diverse populations and larger cohorts is crucial to elucidate the full spectrum of CYP2C8*3’s impact on imatinib metabolism and treatment response.

The ABCG2, also known as the breast cancer resistance protein, belongs to the subfamily G of the ABC efflux transporter superfamily and mediates ATP-dependent efflux of diverse molecules across cell membranes [44]. This protein is encoded by the *ABCG2* gene, which is situated on chromosome 4q22.1 [45].

This transporter is extensively distributed in the human body. It operates in the apical membrane of enterocytes, where it restricts intestinal absorption; in the sinusoidal membrane of hepatocytes, where it facilitates hepatobiliary excretion; and in the apical membrane of proximal tubular cells in the kidney, where it contributes to uric acid elimination [46]. Furthermore, it significantly affects the pharmacokinetics of diverse compounds including anticancer drugs, antibiotics, antivirals and analgesics [47].

Numerous SNPs have been identified in the *ABCG2* gene, with C421A (rs2231142) in exon 5 being extensively studied. This SNP involves a substitution of glutamine with lysine at codon 141 (Q141K) [48].

In both in vitro and in vivo studies, this polymorphism was reported to generally reduce ABCG2 protein expression. Additionally, some studies indicate that it may also diminish ATPase activity, leading to compromised transport function [49].

Given its location on the apical membrane of hepatocytes, ABCG2 is likely essential for the excretion of imatinib. Consequently, genetic variations in the *ABCG2* gene may affect the pharmacokinetics and clinical response to imatinib [18]. However, the precise effect of the *ABCG2* C421A SNP on imatinib’s plasma trough concentration and therapeutic response remains a subject of debate.

In this study, we found that patients with the CA genotype of the *ABCG2* C421A polymorphism showed significantly higher plasma trough concentrations of imatinib compared to those with the CC genotype. Additionally, the CA genotype was more prevalent among responder patients, suggesting a potential role of this polymorphism in enhancing the therapeutic efficacy of imatinib.

Results of the present study align with those of Takahashi et al. [50], who reported higher imatinib trough concentrations in Japanese CML patients with the CA or AA genotypes compared to those with the CC genotype. However, they did not find a significant association between this SNP and treatment response.

Seong and his colleagues [18] identified a potential association between the ABCG2 C421A variant and an increased rate of major molecular response in Korean CML patients. However, they did not observe a significant effect on imatinib trough concentrations.

Jiang et al. [51] conducted a meta-analysis that included seven studies with nearly 2,200 patients, which further reinforced the association between the ABCG2 C421A variant allele and a higher clinical response rate.

Similarly, Alves et al. [52] observed that the CC genotype was associated with imatinib resistance, while the CA genotype was linked to a favorable response in Portuguese CML patients.

Findings of the current study are comparable with those of Cheng et al. [53], who examined the influence of the *ABCG2* C421A polymorphism on imatinib plasma concentration and response in 190 Chinese CML patients. They found that individuals with the CA or AA genotype exhibited higher imatinib concentrations and more favorable cytogenetic and molecular responses compared to those with the CC genotype. This finding supports our observation that the CA genotype is linked to higher plasma trough concentrations of imatinib and an improved therapeutic response.

In contrast, several studies have found no significant association between the *ABCG2* C421A SNP and imatinib response. For instance, Francis et al. [54] reported no significant impact of this SNP on imatinib trough concentration in Indian CML patients.

Similarly, Omran et al. [55] and Rajamani et al. [5] reported no significant influence of the ABCG2 C421A SNP on imatinib response in Egyptian and Indian CML patients, respectively. Additionally, Sabri et al. [56] found no significant association between ABCG2 gene expression and response to imatinib in their study on Egyptian CML patients.

Recent studies by Nouri et al. [57] and Mohammadi et al. [58] also concluded that the ABCG2 C421A SNP had no impact on imatinib response in Iranian CML patients.

The present study faced several limitations. The primary constraint was the small sample size, which might have hindered the identification of some genotypes within the patient population. Thus, large and multicenter studies are necessary. Furthermore, financial restrictions limited our investigation to a selected number of polymorphisms.

## Conclusion

The current study revealed that the *ABCG2* C421A (rs2231142) polymorphism significantly impacted both the plasma trough concentration and molecular response to imatinib in Egyptian patients with chronic-phase CML. Patients carrying the CA genotype showed higher plasma imatinib concentrations and more favorable treatment outcomes compared to those with the CC genotype. In contrast, the CYP2C8*3 (G416A; rs11572080) polymorphism did not significantly affect imatinib pharmacokinetics or clinical outcomes in the study population. These findings suggest that genotyping for the *ABCG2* C421A SNP could be a valuable tool in optimizing imatinib therapy for CML patients, allowing for more personalized treatment strategies.

## Electronic supplementary material

Below is the link to the electronic supplementary material.

Supplementary Material 1

## Data Availability

No datasets were generated or analysed during the current study.
