# Supplementary material for: Influence of CYP2C8*3 and ABCG2 C421A genetic polymorphisms on trough concentration and molecular response of imatinib in Egyptian patients with chronic myeloid leukemia
Source: Cancer Chemother Pharmacol. 2024 Dec 23;95(1):12. doi: 10.1007/s00280-024-04723-y (PMC11666798; doi:10.1007/s00280-024-04723-y)
Supplement: Supplementary file 1 — Supplementary Material 1 [file 280_2024_4723_MOESM1_ESM.pdf]

Influence of *CYP2C8*3* and *ABCG2* C421A genetic polymorphisms on trough concentration and molecular response of imatinib in Egyptian patients with chronic myeloid leukemia

Safwat A. Mangoura[1,2], Mahmoud H. Abdel-Raheem[2], Hanan A. Eltyb[3], Mohammed S. Molla[2], Abeer M. R. Hussein[2]

[1] Department of Pharmacology and Toxicology, Faculty of Pharmacy, Badr University in Cairo (BUC), Badr, Cairo, Egypt

[2] Department of Medical Pharmacology, Faculty of Medicine, Assiut University, Assiut, Egypt

[3] Department of Medical Oncology and Malignant Hematology, South Egypt Cancer Institute (SECI), Assiut University, Assiut, Egypt

Corresponding author: Mohammed S. Molla
Department of Medical Pharmacology, Faculty of Medicine, Assiut University, Assiut 71515, Egypt
E-mail: mohamedsayed@aun.edu.eg
Orchid ID: https://orcid.org/0000-0002-5990-6152

Details of the polymerase chain reaction-restriction fragment length polymorphism (PCR-RFLP) of *CYP2C8*3* and *ABCG2* C421A polymorphisms:

- **a- Polymerase chain reaction amplification of DNA samples:** A 50 µL reaction mixture was prepared in a 0.2 mL PCR tube following the manufacturer's protocol. The mixture included 25 µL of COSMO PCR Master Mix, 2 µL each of forward and reverse primers, 11 µL of nuclease-free water, and 10 µL of the DNA template.
- b- **Restriction fragment length polymorphism analysis**: The amplified PCR products for each gene were digested with the appropriate restriction enzymes according to the manufacturer's protocol. For each sample, a 30 µL restriction reaction mixture was prepared, containing 17 µL of nuclease-free water, 2 µL of buffer, 10 µL of PCR product, and 1 µL of the restriction enzyme.

The primer sequences, PCR thermal conditions, restriction enzymes, and incubation temperatures for each SNP are presented in the following table:

	CYP2C8*3 **(G416A; rs11572080)**	***ABCG2*** **C421A (rs2231142)**
**Primer sequence:**<br>- Forward	5' AGGCAATTCCCCAATATCTC-3'	5'-TGTTGTGATGGGCACTCTGATGTG-3'
- Reverse	5'-ACTCCTCCACAAGGCAGTGA-3'	5'-ATCAGAGTCATTTTATCCACAC -3'
**PCR thermal conditions:**<br>- Initial denaturation	94 °C for 2 minutes.	94 °C for 10 minutes.
- Denaturation	94 °C for 30 seconds	
- Annealing	48 °C for 80 seconds.	53 °C for 30 seconds
- Extension	72 °C for 1 minute.	53 °C for 30 seconds
- Number of cycles	35 cycles	
- Final extension	72 °C for 7 minutes.	
**PCR product [base pair (bp)]**	347	222
**Restriction enzyme**	*Bse*RI	*Hpy*CH4III
**Digestion temperature and duration**	37 °C for 15 minutes	37°C for 1 hours
**Fagments length after digestion (bp):**<br>- Homozygous wild type<br>- Heterozygous type	**GG:** 310.<br>**GA:** 347 and 310.	**CC:** 183.<br>**CA:** 222 and 183

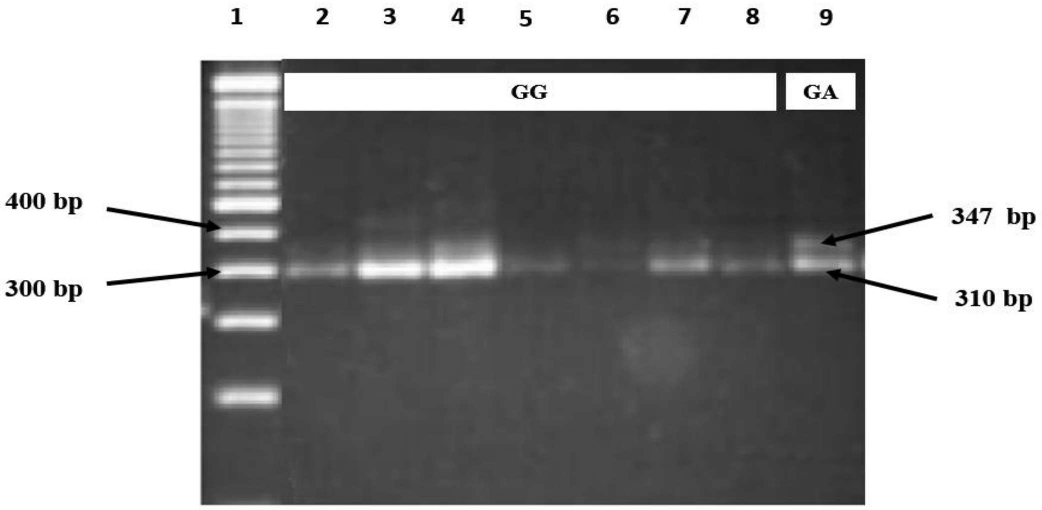

Gel electrophoresis of *CYP2C8*3* (G416A; rs11572080) polymorphism following PCR-RFLP analysis.

Lane 1 contains a 100 bp DNA ladder. Lanes 2–8 display the 310 bp band, indicating the wild-type homozygous genotype (GG; *CYP2C8*1/*1*). Lane 9 shows the 347 bp and 310 bp bands, representing the heterozygous genotype (GA; *CYP2C8*1/*3*).

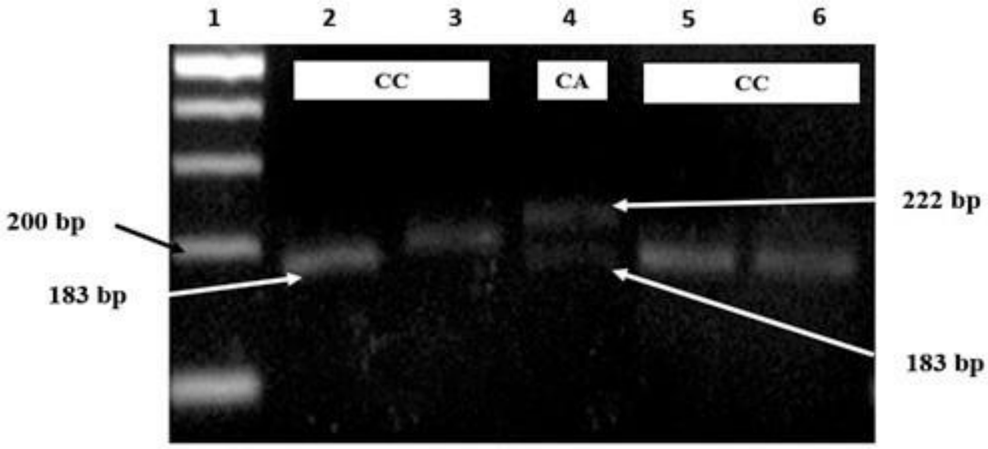

Gel electrophoresis of *ABCG2* C421A (rs2231142) polymorphism following PCR-RFLP analysis.

Lane 1 contains a 100 bp DNA ladder. Lanes 2, 3, 5, and 6 display the 183 bp band, indicating the wild-type homozygous genotype (CC). Lane 4 shows both the 222 bp and 183 bp bands, representing the heterozygous genotype (CA).
